# What Personal and Work-Related Characteristics of Dutch Construction Workers With Knee Osteoarthritis Are Associated With Future Work Ability?

**DOI:** 10.1097/JOM.0000000000002730

**Published:** 2022-10-13

**Authors:** Britte L. De Kock, Jack Van der Gragt, Henk F. Van der Molen, P. Paul F.M. Kuijer, Nina Zipfel

**Affiliations:** From the Department of Public and Occupational Health, Coronel Institute of Occupational Health, Amsterdam University Medical Center, University of Amsterdam, Amsterdam Public Health Research Institute, Amsterdam, The Netherlands (Ms De Kock, and Drs Van der Molen, Kuijer, and Zipfel); Volandis, Harderwijk, The Netherlands (Dr Van der Gragt); Societal Participation & Health, Quality of Care, Amsterdam Public Health Research Institute, Amsterdam, The Netherlands (Drs Van der Molen and Kuijer); and Musculoskeletal Health and Sports, Amsterdam Movement Sciences, Amsterdam, The Netherlands (Dr Kuijer).

**Keywords:** prognosis, work ability, knee osteoarthritis, physical activity, prevention, occupational health surveillance

## Abstract

To assess personal and work-related characteristics of construction workers with knee osteoarthritis (KO) associated with their ability to perform their current profession in the following 2 years.

**Methods:**

A cross-sectional study was performed among Dutch construction workers diagnosed with KO using data from the Worker Health Surveillance. Logistic regression was used to assess the characteristics associated with future work ability.

**Results:**

On the basis of 344 construction workers with KO, being able to perform their current profession in 2 years' time was associated with working weekly 36 to 45 hours (odds ratio [OR], 3.0 to 6.3), performing high-intensity exercises 1 to 2 times weekly (OR, 2.0 to 2.6), being younger than 56 years (OR, 0.2 to 0.3), and not performing strenuous work activities such as lifting and kneeling (OR, 0.4 to 0.5).

**Conclusions:**

To keep construction workers with KO at work, intervention studies should evaluate the effects of reducing strenuous work activities and promote leisure-time exercise.

## Abstract 2

In the upcoming decade, the prevalence of knee osteoarthritis will rise steeply, especially among workers. Therefore, a better insight in prognostic factors to maintain future work ability is of importance, especially in physically demanding jobs. This study shows that both occupational and leisure-time physical activity needs to be addressed.

The construction industry is characterized by high physical work demands and prevalent work-related musculoskeletal disorders.^1–3^ A review including a meta-analysis showed that knee complaints are the most common complaint in the construction industry second only to low-back pain, with a 1-year prevalence of 37% (95% confidence interval [CI], 22% to 52%).^4^ These knee complaints are in large part attributable to knee osteoarthritis (KO), given the high prevalence of this work-related disease compared with other diagnoses such as meniscal tears or bursitis.^5–7^ In the upcoming years, a steep rise in KO is forecasted, especially among workers aged between 40 and 65 years.^8^ Specific construction professions with a high risk of KO are floor layers, asphalt workers, sheet-metal workers, rock workers, plumbers, bricklayers, wood workers, and concrete workers.^9^

Given the high prevalence of KO and the high physical work demands, these construction workers deserve timely and effective occupational health care to maintain their work ability despite the debilitating effect of KO. Doctor-diagnosed KO has an almost twofold increased risk of sick leave and about 40% to 50% increased risk of disability pension compared with the general population.^10^ In addition, a recent Dutch study showed that the annual sick leave costs due to KO in the Dutch workforce are substantial for workers consulting an occupational physician, with estimated costs of $43 million. The average costs per sick leave episode are also considerable—$16,846 for KO with an average sick leave duration of 186 days.^11^ Unfortunately, no occupational health guidelines are available that address how work ability among workers with KO should be managed. Present clinical guidelines for nonoperative treatment are primarily focused on pain and self-reported function of activities of daily life.^12^ These outcomes are not always a good proxy for being able to work.^13^

To prevent sick leave and early retirement, and to contribute to a life span approach for KO, occupational health care should target prognostic factors that enhance the construction workers' future work ability.^14^ Therefore, the aim of this study is to assess the personal and work-related characteristics of construction workers diagnosed with KO associated with their ability to perform their current profession in the following 2 years.

## METHODS

### Study Design

A cross-sectional study was conducted to determine the association between personal and work-related characteristics and the self-reported future ability of construction workers with KO to practice their current profession in 2 years' time. The Strengthening the Reporting of Observational Studies in Epidemiology (STROBE)checklist was used to describe this cross-sectional study (Appendix 1, http://links.lww.com/JOM/B219).^15^

### Participants and Measurement

For this study, male construction workers aged between 20 and 68 years were selected. Participants were included when they received a KO diagnosis by an occupational physician. Data were used from the Worker Health Surveillance (WHS) for construction workers performed between 2011 and 2021 in the Netherlands. The WHS consists of two parts, consisting of (1) a standardized physical examination and (2) a self-administered questionnaire. Construction workers were invited to participate in the WHS starting at the age of 20 years, followed by periodic checkups every 5 years and more regularly after the age of 50 years.

### Variables

For the purpose of this study, specific questions from the WHS were selected focusing on knee complaints and future work ability (Appendix 2, http://links.lww.com/JOM/B220). Questions were selected based on risk factors from the literature for KO and work ability.^9,10,16–33^

### Study Size

**FIGURE 1:**
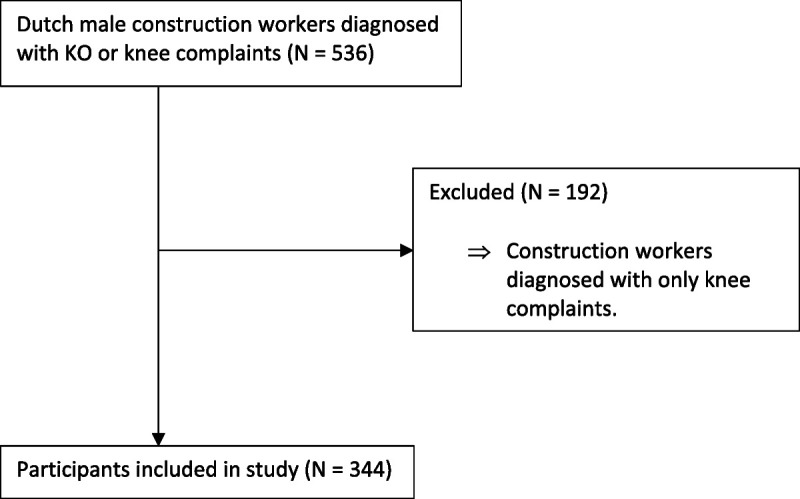
Flowchart: inclusion and exclusion criteria of study participants.

For this study, all construction workers were included if doctor-diagnosed KO was established. Data from the WHS between 2011 and 2021 were used, which resulted in a total sample size of 344 male construction workers with clinically diagnosed KO (Fig. 1).

### Primary Outcomes

The selected primary outcome was the self-reported prognosis of work ability to practice the current profession in 2 years' time measured with a self-administered questionnaire taken from the Work Ability Index questionnaire.^34,35^ The answering categories were unlikely, maybe, and very likely. For this study, the population was divided into two groups: (1) construction workers who reported being “very likely to be practicing their current profession in 2 years' time” (answering category: “very likely”) and (2) construction workers who reported being “not very likely to be practicing their current profession in 2 years' time” (answering categories: “maybe” and “unlikely”). The two groups were created to form comparable group sizes in the number of participants. Independent variables were age, body mass index (BMI; body height and body weight were measured during the physical examination), number of years in the current profession, average number of working hours per week, number of times per week of high-intensity exercise, performance of strenuous work activities, and the worker being of the opinion that work caused the complaints. Continuous variables were recoded into categories. Age was recoded into four categories (<40 to 49 years, 50 to 55 years, 56 to 60 years, >60 years). Given the increased risk of KO with older age, in our opinion, this categorization best represented the included participant group. Body mass index was categorized as normal weight (BMI of 18 to 25 kg/m^2^), overweight (BMI of 25 to 30 kg/m^2^), and obesity (BMI > 30 kg/m^2^). Number of years in the current profession was categorized as 0 to 10 years, 11 to 20 years, 21 to 30 years, 31 to 40 years, and 41 to 50 years. Average number of working hours per week was categorized as 3 to 35 hours, 36 to 40 hours, 41 to 45 hours, and >45 hours. Number of times per week of high-intensity exercise was categorized as 0 times, 1 time, 2 times, and >3 times. Performing regularly strenuous work activities was defined as (1) bending over, (2) kneeling or squatting, and/or (3) lifting, pushing, pulling, or carrying heavy loads during work, with the answering categories of no strenuous work activity, one of these strenuous work activities, two of these activities, and three of these activities. Finally, work ability on a scale from 0 to 10 was categorized as 0 to 5 being poor, 6 to 7 being moderate, 8 to 9 being good, and 10 being excellent.

### Statistical Methods

To get insight into the association between personal and work-related characteristics and the future ability to practice the current profession in 2 years' time, a binary logistic regression was conducted. First, a univariable analysis was conducted. Second, a multivariable binary logistic regression model was performed based on the significance of variables set at *P* ≤ 0.05 from the univariable analysis. For the multivariable regression model, the forward stepwise procedure was used. Before performing the regression analysis, the included variables were tested on collinearity and power (Appendix 3, http://links.lww.com/JOM/B221). The quality of the model was assessed with the −2-log likelihood ratio test and the Hosmer-Lemeshow test (Appendix 4, http://links.lww.com/JOM/B222, and 5, http://links.lww.com/JOM/B223). A Hosmer-Lemeshow chi-squared with *P* ≥ 0.05 showed goodness of fit. For the results, categorical variables were presented as numbers and percentages. Numerical variables were additionally presented as mean and standard deviation. The results of the logistic regression analysis were presented using odds ratio (OR) and their corresponding 95% CI, and the *P* value was set at *P* ≤ 0.05. Data were analyzed using IBM SPSS Statistics version 26.

## RESULTS

### Participants and Descriptive Data

As already stated, a total of 344 Dutch construction workers diagnosed with KO were included in this study (Fig. 1). The personal and work-related characteristics of the participants are presented in Table 1. Most of the participants were above the age of 56 years, felt pain or stiffness in their knee, were overweight, and performed high-intensity exercise zero times per week. Moreover, they worked 36 to 40 hours per week and had worked in their current profession for 21 to 30 years. Participants reported having good physical and mental work ability and reported regularly bending over, kneeling or squatting, and lifting, pushing, pulling, or carrying heavy loads during work.

**TABLE 1:** Descriptive Personal and Work-Related Characteristics of Construction Workers With KO

	*n* (%)	Mean (SD)
Personal characteristics		
Age, yrs		56.5 (6.1)
<40–49	47 (14)	
50–55	74 (22)	
56–60	119 (25)	
>60	104 (30)	
Pain or stiffness in knee the previous year		
No	42 (12)	
Yes	301 (88)	
No. times per week of high-intensity exercise (eg, soccer, running, indoor sports, physically demanding work)		2.3 (1.2)
0	120 (40)	
1	40 (13)	
2	85 (28)	
>3	59 (19)	
BMI		28.3 (3.7)
Normal weight (BMI of 18–25)	62 (18)	
Overweight (BMI of 25–30)	186 (54)	
Obesity (BMI > 30)	96 (28)	
Work-related characteristics		
Average no. workhours per week		39.9 (7.2)
3–35	53 (15)	
36–40	207 (60)	
41–45	43 (13)	
>45	40 (12)	
No. years working in the current profession		24.8 (13.1)
0–10	64 (19)	
11–20	68 (20)	
21–30	95 (28)	
31–40	65 (19)	
41–50	51 (15)	
Work ability at the moment (physical and mental)		7.3 (1.4)
0–5, poor	30 (9)	
6–7, moderate	143 (42)	
8–9, good	150 (44)	
10, excellent	17 (5)	
Strenuous work activities: (1) bending over, (2) kneeling or squatting, and (3) lifting, pushing, pulling, or carrying heavy loads during work		1.3 (1.1)	
No	99 (30)		
One work activity	86 (26)		
Two work activities	77 (24)		
Three work activities	64 (20)		

BMI, body mass index; KO, knee osteoarthritis; SD, standard deviation.

### Personal and Work-Related Characteristics of Work Ability in 2 Years' Time

The univariable binary logistic regression was performed first for the seven selected variables separately (Table 2). The two variables of BMI and whether work had caused complaints were not significantly associated with work ability in 2 years' time (*P* = 0.42 and *P* = 0.55, respectively) and were thus not included in the multivariable analysis.

**TABLE 2:** Univariate Analysis of the Association (Odds Ratio, 95% Confidence Interval, *P* Value) Between Personal and Work-Related Characteristics and Ability to Practice the Current Profession in 2 Years' Time

	Odds Ratio	95% Confidence Interval	*P* Value
Personal characteristics			
Age, yrs			
(Ref: <40–49)	1.00		0.000
50–55	0.85	0.73–1.94	0.699
56–60	0.34	0.16–0.71	0.004
>60	0.25	0.12–0.54	0.000
No. times per week of high-intensity exercise (eg, soccer, running, indoor sports, physically demanding work)			
(Ref: 0)	1.00		0.009
1	2.54	1.20–5.39	0.02
2	2.24	1.27–3.97	0.006
>3	1.10	0.59–2.06	0.756
BMI			
(Ref: normal weight [BMI of 18–25])	1.00		0.42
Overweight (BMI of 25–30)	0.68	0.38–1.23	0.199
Obesity (BMI > 30)	0.697	0.36–1.34	0.28
Work-related characteristics			
I can do my work, but it does cause some complaints			
(Ref: No)	1.00		0.55
Yes	0.88	0.57–1.35	0.55
Average no. work hours per week			
(Ref: 3–35)	1.00		0.001
36–40	2.58	1.37–4.85	0.003
41–45	6.42	2.59–15.90	0.000
>45	2.15	0.93–4.90	0.08
No. years working in the current profession			
(Ref: 0–10)	1.00		0.003
11–20	1.53	0.75–3.10	0.24
21–30	0.88	0.47–1.67	0.70
31–40	1.095	0.54–2.21	0.80
41–50	0.34	0.15–0.72	0.005
Strenuous work activities: (1) bending over, (2) kneeling or squatting, and (3) lifting, pushing, pulling, or carrying heavy loads during work			
(Ref: no)	1.00		0.02
One work activity	1.04	0.58–1.89	0.89
Two work activities	0.63	0.35–1.16	0.14
Three work activities	0.42	0.22–0.79	0.008

BMI, body mass index; Ref, reference.

The final model for the analysis on the association between personal and work-related characteristics on work ability in 2 years' time is presented in Table 3 and included four variables. Personal and work-related characteristics associated with the ability to practice the current profession in 2 years' time were 1-time high-intensity exercise per week (OR, 2.62; 95% CI, 1.12 to 6.15), 2 times per week of high-intensity exercise (OR, 2.00; 95% CI, 1.06 to 3.80), working for 36 to 40 hours per week (OR, 3.01; 95% CI, 1.36 to 6.67), working 41 to 45 hours per week (OR, 6.25; 95% CI, 2.06 to 18.99), performing two strenuous work activities (OR, 0.45; 95% CI, 0.22 to 0.95), performing three strenuous work activities (OR, 0.40; 95% CI, 0.18 to 0.88), age between 56 and 60 years (OR, 0.22; 95% CI, 0.09 to 0.54), and age > 60 years (OR, 0.28; 95% CI, 0.11 to 0.69). The variable numbers of years working in the current profession was not significant and was thus not included in the final model. The Hosmer-Lemeshow test showed a goodness of fit (χ^2^ = 4.538, *P* = 0.806).

**TABLE 3:** Multivariable Analysis of the Association (Odds Ratio, 95% Confidence Interval, *P* Value) Between Personal and Work-Related Characteristics and Ability to Practice the Current Profession in 2 Years' Time

	*N* Total (*N* = 343)	Construction Workers Who Are Very Likely to Still Practice Their Current Function in 2 Years' Time (*N* = 190)	Construction Workers Who Are Unlikely or Maybe Able to Practice Their Current Function in 2 Years' Time (*N* = 153)	Odds Ratio	95% Confidence Interval	*P* Value
Personal characteristics						
Age, yrs						
(Ref: <40–49)	47	35	12	1.00		
50–55	74	53	21	0.74	0.28–1.93	0.53
56–60	119	59	60	0.22	0.09–0.54	0.001
>60	104	44	60	0.28	0.11–0.69	0.006
No. times per week of high-intensity exercise (eg, soccer, running, indoor sports, physically demanding work)						
(Ref: 0)	120	54	66	1.00		
1	40	27	13	2.62	1.12–6.15	0.03
2	85	55	30	2.00	1.06–3.80	0.03
>3	59	28	31	1.10	0.50–2.31	0.87
Work-related characteristics						
Average no. work hours per week						
(Ref: 3–35)	53	18	35	1.00		
36–40	207	118	89	3.01	1.36–6.67	0.007
41–45	43	33	10	6.25	2.06–18.99	0.001
>45	40	21	19	1.61	0.58–4.45	0.36
Strenuous work activities: (1) bending over, (2) kneeling or squatting, and (3) lifting, pushing, pulling, or carrying heavy loads during work						
(Ref: no)	102	62	40	1.00		
One work activity	87	53	34	1.12	0.56–2.23	0.76
Two work activities	72	34	38	0.45	0.22–0.95	0.04
Three work activities	65	27	38	0.40	0.18–0.88	0.02

Ref, reference.

## DISCUSSION

Our study found that the ability to practice the current profession in 2 years' time is associated with high-intensity exercises once or twice a week, working for 36 to 45 hours per week, performing less than two strenuous work activities, and being younger than 56 years.

The following findings seem in line with previous studies on the topic of having KO and enhancing work participation. For instance, being physically active outside work seems especially important for workers in physically strenuous jobs. Despite that the guidelines of the World Health Organization on physical activity and sedentary behavior make no distinction between occupational physical activity and leisure-time physical activity, the present results support the assumption that the so-called Goldilocks Principle also applies for workers with KO.^36^ This is in line with the results of a recent evaluation of this principle for construction and health care workers, reporting that workers who spent more time on physical activity during leisure reported less musculoskeletal pain.^37^ Of course, being physically active in leisure time is easier said than done given that construction workers do not have a fixed workplace and therefore often have to travel back and forth from work, leaving little time and energy to exercise before or after work. The positive work ability results for remaining physically active in leisure time are also in line with the nonoperative treatment guidelines for KO, supporting a physically active lifestyle to enhance participation.^38^ Moreover, the Good Life with osteoArthritis in Denmark (GLA:D™) (Research Unit for Musculoskeletal Function and Physiotherapy at the University of Southern Denmark) showed that regular intense exercise (twice weekly for 6 weeks) seemed to improve work participation, reducing sickness absence in 1 year from 24% (95% CI, 21 to 28) to 15% (95% CI, 12 to 18).^39^

Not surprisingly, a high physical workload due to frequent bending over, kneeling or squatting, and lifting, pushing, pulling, or carrying heavy loads is not beneficial for the work ability of construction workers with KO. A high physical workload is an established risk factor for the onset or worsening of KO.^14,33^ Moreover, KO limits the ability to perform these activities, and therefore, the negative association found with the future work ability of construction workers with KO is in line with the literature.^29^ The same line of reasoning applies for older age. The prevalence of KO is of course strongly related to an increase of age.^14,17^ In our study, we decided to create a composite outcome variable of physical workload activities as we expect a high correlation between these activities.^30^ However, we also conducted an additional analysis of the physical workload activities separately (Appendix 6, http://links.lww.com/JOM/B224). The results of the additional analysis showed that the work activity of bending over of the trunk in the univariate analysis was not found to be significant. This might also be expected given that KO is less likely to limit trunk activity compared with the other two more strongly associated activities that are more demanding for the lower extremities, namely, “kneeling or squatting” and “lifting, pushing, pulling, or carrying heavy loads during work.”

Not all findings seem in line with previous studies, however. The fact that working less than 36 hours per week does not support future work ability seems contradictory to current findings.^40^ Working 36 to 45 hours seems optimal. However, continuing to work a high number of hours as a construction worker does not seem a wise decision, despite construction workers not being able to resist it. On the other hand, the result in this study can possibly be explained due to a *healthy worker's survival effect*, which means that, in this study, construction workers with KO who were absent from work or who retired early did not participate in the surveillance.^41^ However, we believe that the risk of selection bias is minimal in our study.^42^ Both the two oldest age groups (56 to 60 years and >60 years) are less likely to do their current profession in 2 years' time compared with their younger colleagues (see Table 3). This is probably explained by the fact that KO is a progressive disease for which no cure is available.^16,17^

It is interesting to note that BMI was not found to be associated with a less favorable prognosis of work ability in 2 years' time.^43^ This is counterintuitive given that BMI is associated with an increased risk of KO^14^ and having a higher BMI as a KO patient results in a higher pain score.^44^ A possible explanation is that workers with KO and being overweight or obese have already structured their job in such a manner that they no longer need to perform the most demanding activities, given their body weight. Further research should be conducted to properly determine the influence of BMI on the prospects of the further career of the construction workers with KO. Moreover, the use of BMI has been debated in the literature as controversial.^45^ Although BMI might not be the most accurate and suitable measure for determining overweight or obesity, it is one of the most used measurements to screen overweight or obesity risks in various population groups and is well suitable for epidemiological settings such as the correlation between health outcomes and BMI.^46^ Therefore, measurement of BMI was deemed appropriate for the purpose of the current study, but future studies should investigate the influence of body composition on future work ability especially in physically demanding jobs.

To offer tailor-made prevention and interventions for male construction workers with KO, future research should focus on reducing physically demanding work activities and promoting high-intensity exercise for at least once a week. Previous research among construction workers showed that health promotion worksite interventions improved physical activity and may, thus, contribute to a higher ability to practice their current profession in the future.^47^ In addition, a Total Worker Health® intervention among construction workers showed that, at 6 months, the intervention group had reduced physically demanding activities and increased recreational physical activity.^48^ Unfortunately, limited evidence is available to support the suggestion that the use of preventive ergonomic measures to reduce the workload actually reduces the risk of associated musculoskeletal complaints.^49^ However, health impact assessments based on worksite measurements potentially show positive gains of ergonomics measures at the worksite among construction workers at risk of KO.^50^

### Limitations and Strengths

A major strength of our study is the presence of a high number of construction workers diagnosed with KO by an occupational physician. Moreover, investigating the combination of personal and work-related characteristics can also be seen as a strength, because both factors influence the ability to continue working as a construction worker with KO. Finally, work ability is measured using a validated method.^24^

Nevertheless, we faced three limitations in our study. First, this study had a cross-sectional design that does not allow investigating the causal relationship between the outcome and variables.^51^ Second, the method of diagnosing KO used by the various occupational physicians might have differed. This could have resulted in a more heterogeneous selection of workers who did not have KO as the primary cause of their knee complaints. Moreover, participation in the WHS is voluntary, which might have resulted in a healthy worker selection effect.^41^ Reasons for not participating or having a lower commitment could be that construction workers were afraid that a negative outcome regarding their future career opportunities might result, or they did not see the personal relevance to participate.^41^ A third limitation was that the WHS questionnaire made no explicit distinction between occupational physical activity and leisure-time physical activity.^52^ This may have introduced classification bias as it is challenging to distinguish whether exercise concerns occupational physical activity or leisure-time physical activity.^52^ An earlier study recommends physical activity assessment based on objective measures including, for example, accelerometers instead of self-reported questionnaire-based assessment.^52^ However, on the basis of the results of the current study, we assume that participants considered the question on exercise as leisure-time physical activity as most participants reported zero times per week of high-intensity exercise.

Finally, for the primary outcome measure of the self-reported prognosis of work ability to practice the current profession in 2 years' time, the categories “maybe” and “unlikely” were combined to form a dichotomous outcome measure for the analysis. A combination of those categories might be conservative, but we aimed to study the prognostic factors that enhance the construction workers' future work ability to prevent sick leave and early retirement targeting the group that may be at risk of being unable to do their current profession in 2 years' time and that believes to be certain to be unable to do their current profession in 2 years' time. Thus, the current study gives insight into the needs for tailoring future interventions to the prognostic factors of both the at-risk group and the group unlikely to do their current profession in the future.

## CONCLUSION

Dutch construction workers with clinically diagnosed KO who reported that, in 2 years' time, they would be able to practice their current job were working 36 to 45 hours per week, performing high-intensity exercises once or twice a week, were younger than 56 years, and performed less than two strenuous physical activities in their work compared with their counterparts who reported not being able to practice their current job in 2 years' time. To keep construction workers with KO at work, intervention studies should evaluate the effects of reducing strenuous work activities and promote leisure-time exercises.

## Supplementary Material

SUPPLEMENTARY MATERIAL

## References

[1] Colin R Wild P Paris C Boini S. Effect of joint exposure to psychosocial and physical work factors on the incidence of workplace injuries: results from a longitudinal survey. *J Occup Environ Med*. 2021;63:921–930. 34238905 10.1097/JOM.0000000000002313

[2] What are the risks to minors who work in the construction industry? *J Occup Environ Med*. 2021;63:e462–e463. 34029298 10.1097/JOM.0000000000002273 PMC10141679

[3] Dale AM Rohlman DS Hayibor L Evanoff BA. Work organization factors associated with health and work outcomes among apprentice construction workers: comparison between the residential and commercial sectors. *Int J Environ Res Public Health*. 2021;18:8899. 34501489 10.3390/ijerph18178899 PMC8430912

[4] Umer W Antwi-Afari MF Li H Szeto GPY Wong AYL. The prevalence of musculoskeletal symptoms in the construction industry: a systematic review and meta-analysis. *Int Arch Occup Environ Health*. 2018;91:125–144. 29090335 10.1007/s00420-017-1273-4

[5] Hulshof CTJ Pega F Neupane S, . The effect of occupational exposure to ergonomic risk factors on osteoarthritis of hip or knee and selected other musculoskeletal diseases: a systematic review and meta-analysis from the WHO/ILO joint estimates of the work-related burden of disease and injury. *Environ Int*. 2021;150:106349. 33546919 10.1016/j.envint.2020.106349

[6] Bahns C Bolm-Audorff U Seidler A Romero Starke K Ochsmann E. Occupational risk factors for meniscal lesions: a systematic review and meta-analysis. *BMC Musculoskelet Disord*. 2021;22:1042. 34911509 10.1186/s12891-021-04900-7 PMC8672613

[7] Le Manac'h AP Ha C Descatha A Imbernon E Roquelaure Y. Prevalence of knee bursitis in the workforce. *J Occup Environ Med*. 2012;62:658–660. 10.1093/occmed/kqs113 22778241

[8] Kuijer P Burdorf A. Prevention at work needed to curb the worldwide strong increase in knee replacement surgery for working-age osteoarthritis patients. *Scand J Work Environ Health*. 2020;46:457–460. 32780145 10.5271/sjweh.3915 PMC7737795

[9] Jarvholm B From C Lewold S Malchau H Vingard E. Incidence of surgically treated osteoarthritis in the hip and knee in male construction workers. *J Occup Environ Med*. 2008;65:275–278. 10.1136/oem.2007.033365 17928390

[10] Hubertsson J Petersson IF Thorstensson CA Englund M. Risk of sick leave and disability pension in working-age women and men with knee osteoarthritis. *Ann Rheum Dis*. 2013;72:401–405. 22679305 10.1136/annrheumdis-2012-201472

[11] Hardenberg M Speklé EM Coenen P Brus IM Kuijer PPFM. The economic burden of knee and hip osteoarthritis: absenteeism and costs in the Dutch workforce. *BMC Musculoskelet Disord*. 2022;23:364. 35436874 10.1186/s12891-022-05306-9 PMC9017043

[12] Bannuru RR Osani MC Vaysbrot EE, . OARSI guidelines for the non-surgical management of knee, hip, and polyarticular osteoarthritis. *Osteoarthr Cartil*. 2019;27:1578–1589. 10.1016/j.joca.2019.06.011 31278997

[13] Van Zaanen Y Hoorntje A Koenraadt KLM, . Non-surgical treatment before hip and knee arthroplasty remains underutilized with low satisfaction regarding performance of work, sports, and leisure activities. *Acta Orthop*. 2020;91:717–723. 32878525 10.1080/17453674.2020.1813440 PMC8023969

[14] Whittaker JL Runhaar J Bierma-Zeinstra S Roos EM. A lifespan approach to osteoarthritis prevention. *Osteoarthr Cartil*. 2021;29:1638–1653. 10.1016/j.joca.2021.06.015 34560260

[15] STROBE. STROBE checklists. Available at: https://www.strobe-statement.org/checklists/. Accessed October 16, 2007.

[16] Katz JN Arant KR Loeser RF. Diagnosis and treatment of hip and knee osteoarthritis: a review. *JAMA*. 2021;325:568–578. 33560326 10.1001/jama.2020.22171 PMC8225295

[17] Shane Anderson A Loeser RF. Why is osteoarthritis an age-related disease? *Best Pract Res Clin Rheumatol*. 2010;24:15–26. 20129196 10.1016/j.berh.2009.08.006 PMC2818253

[18] Altman R Asch E Bloch D, . Development of criteria for the classification and reporting of osteoarthritis. Classification of osteoarthritis of the knee. Diagnostic and Therapeutic Criteria Committee of the American Rheumatism Association. *Arthritis Rheum*. 1986;29:1039–1049. 3741515 10.1002/art.1780290816

[19] Stubbs B Aluko Y Myint PK Smith TO. Prevalence of depressive symptoms and anxiety in osteoarthritis: a systematic review and meta-analysis. *Age Ageing*. 2016;45:228–235. 26795974 10.1093/ageing/afw001

[20] Toivanen AT Heliövaara M Impivaara O, . Obesity, physically demanding work and traumatic knee injury are major risk factors for knee osteoarthritis—a population-based study with a follow-up of 22 years. *Rheumatology*. 2009;49:308–314. 19946021 10.1093/rheumatology/kep388

[21] Wilkie R Blagojevic-Bucknall M Jordan KP Lacey R McBeth J. Reasons why multimorbidity increases the risk of participation restriction in older adults with lower extremity osteoarthritis: a prospective cohort study in primary care. *Arthritis Care Res (Hoboken)*. 2013;65:910–919. 23225783 10.1002/acr.21918

[22] Connelly AE Tucker AJ Kott LS Wright AJ Duncan AM. Modifiable lifestyle factors are associated with lower pain levels in adults with knee osteoarthritis. *Pain Res ManagPain Research and Management*. 2015;20:241–248. 10.1155/2015/389084 PMC4596631 26125195

[23] Zheng H Chen C. Body mass index and risk of knee osteoarthritis: systematic review and meta-analysis of prospective studies. *BMJ Open*. 2015;5:e007568. 10.1136/bmjopen-2014-007568 PMC4679914 26656979

[24] Łastowiecka E Bugajska J Najmiec A Rell-Bakalarska M Bownik I Jędryka-Góral A. Occupational work and quality of life in osteoarthritis patients. *Rheumatol Int*. 2006;27:131–139. 17094005 10.1007/s00296-006-0177-5

[25] Kujala UM Kettunen J Paananen H, . Knee osteoarthritis in former runners, soccer players, weight lifters, and shooters. *Arthritis Rheum*. 1995;38:539–546. 7718008 10.1002/art.1780380413

[26] Jarvholm B Lewold S Malchau H Vingard E. Age, bodyweight, smoking habits and the risk of severe osteoarthritis in the hip and knee in men. *Eur J Epidemiol*. 2005;20:537–542. 16121763 10.1007/s10654-005-4263-x

[27] Holmberg S Thelin A Thelin N. Is there an increased risk of knee osteoarthritis among farmers? A population-based case-control study. *Int Arch Occup Environ Health*. 2004;77:345–350. 15127209 10.1007/s00420-004-0518-1

[28] Andersen S Thygesen LC Davidsen M Helweg-Larsen K. Cumulative years in occupation and the risk of hip or knee osteoarthritis in men and women: a register-based follow-up study. *J Occup Environ Med*. 2012;69:325–330. 10.1136/oemed-2011-100033 22241844

[29] McWilliams DF Leeb BF Muthuri SG Doherty M Zhang W. Occupational risk factors for osteoarthritis of the knee: a meta-analysis. *Osteoarthritis Cartilage*. 2011;19:829–839. 21382500 10.1016/j.joca.2011.02.016

[30] Manninen P Heliovaara M Riihimaki H Suoma-Iainen O. Physical workload and the risk of severe knee osteoarthritis. *Scand J Work Environ Health*. 2002;28:25–32. 11871849 10.5271/sjweh.643

[31] Jones GT Harkness EF Nahit ES McBeth J Silman AJ Macfarlane GJ. Predicting the onset of knee pain: results from a 2-year prospective study of new workers. *Ann Rheum Dis*. 2007;66:400–406. 16935910 10.1136/ard.2006.057570 PMC1856001

[32] Kievit AJ van Geenen RC Kuijer PP Pahlplatz TM Blankevoort L Schafroth MU. Total knee arthroplasty and the unforeseen impact on return to work: a cross-sectional multicenter survey. *J Arthroplasty*. 2014;29:1163–1168. 24524779 10.1016/j.arth.2014.01.004

[33] Verbeek J Mischke C Robinson R, . Occupational exposure to knee loading and the risk of osteoarthritis of the knee: a systematic review and a dose-response meta-analysis. *Saf Health Work*. 2017;8:130–142. 28593068 10.1016/j.shaw.2017.02.001 PMC5447410

[34] Ilmarinen J. The work ability index (WAI). *J Occup Environ Med*. 2007;57:160.

[35] de Zwart BC Frings-Dresen MH van Duivenbooden JC. Test-retest reliability of the work ability index questionnaire. *Occup Med (Lond)*. 2002;52:177–181. 12091582 10.1093/occmed/52.4.177

[36] World Health Organization. *WHO Guidelines on Physical Activity and Sedentary Behaviour*. Geneva, Switzerland: World Health Organization; 2020.

[37] Merkus SL Coenen P Forsman M Knardahl S Veiersted KB Mathiassen SE. An exploratory study on the physical activity health paradox—musculoskeletal pain and cardiovascular load during work and leisure in construction and healthcare workers. *Int J Environ Res Public Health*. 2022;19:2751. 35270444 10.3390/ijerph19052751 PMC8910306

[38] Jayabalan P Ihm J. Rehabilitation strategies for the athletic individual with early knee osteoarthritis. *Curr Sports Med Rep*. 2016;15:177–183. 27172082 10.1249/JSR.0000000000000260 PMC6784825

[39] Skou ST Roos EM. Good life With osteoArthritis in Denmark (GLA:D™): evidence-based education and supervised neuromuscular exercise delivered by certified physiotherapists nationwide. *BMC Musculoskelet Disord*. 2017;18:72. 28173795 10.1186/s12891-017-1439-y PMC5297181

[40] Iliades C. *Managing Osteoarthritis at Work*. New York, NY: Everyday Health Group; 2009.

[41] Siebert U Rothenbacher D Daniel U Brenner H. Demonstration of the healthy worker survivor effect in a cohort of workers in the construction industry. *J Occup Environ Med*. 2001;58:774–779. 10.1136/oem.58.12.774 PMC1740083 11706143

[42] Chowdhury R Shah D Payal AR. Healthy worker effect phenomenon: revisited with emphasis on statistical methods—a review. *Indian J Occup Environ Med*. 2017;21:2–8. 29391741 10.4103/ijoem.IJOEM_53_16 PMC5763838

[43] Robroek SJW Järvholm B van der Beek AJ Proper KI Wahlström J Burdorf A. Influence of obesity and physical workload on disability benefits among construction workers followed up for 37 years. *J Occup Environ Med*. 2017;74:621–627. 10.1136/oemed-2016-104059 28391246

[44] Raud B Gay C Guiguet-Auclair C, . Level of obesity is directly associated with the clinical and functional consequences of knee osteoarthritis. *Sci Rep*. 2020;10:3601. 32107449 10.1038/s41598-020-60587-1 PMC7046749

[45] Frankenfield DC Rowe WA Cooney RN Smith JS Becker D. Limits of body mass index to detect obesity and predict body composition. *Nutrition*. 2001;17:26–30. 11165884 10.1016/s0899-9007(00)00471-8

[46] Claessen H Arndt V Drath C Brenner H. Overweight, obesity and risk of work disability: a cohort study of construction workers in Germany. *Occup Environ Med*. 2009;66:402–409. 19196736 10.1136/oem.2008.042440

[47] Viester L Verhagen EALM Bongers PM van der Beek AJ. The effect of a health promotion intervention for construction workers on work-related outcomes: results from a randomized controlled trial. *Int Arch Occup Environ Health*. 2015;88:789–798. 25481382 10.1007/s00420-014-1007-9

[48] Peters SE Grant MP Rodgers J Manjourides J Okechukwu CA Dennerlein JT. A cluster randomized controlled trial of a Total Worker Health® intervention on commercial construction sites. *Int J Environ Res Public Health*. 2018;15:2354. 30366387 10.3390/ijerph15112354 PMC6265748

[49] van der Molen HF Sluiter JK Frings-Dresen MH. The use of ergonomic measures and musculoskeletal complaints among carpenters and pavers in a 4.5-year follow-up study. *Ergonomics*. 2009;52:954–963. 19629810 10.1080/00140130902763560

[50] Visser S Van der Molen H Kuijer P. A health impact assessment of a preventive measure to reduce the risk of work-related low back pain, lumbosacral radiculopathy and knee osteoarthritis among construction workers in the Netherlands. *Saf Health Work*. 2022;13:S145. 10.3390/ijerph20054672 PMC10001867 36901682

[51] Kestenbaum B. *Cross-Sectional Studies. Epidemiology and Biostatistics: Practice Problem Workbook*. Cham, Switzerland: Springer International Publishing; 2019:9–11.

[52] Coenen P Huysmans MA Holtermann A, . Towards a better understanding of the ‘physical activity paradox’: the need for a research agenda. *Br J Sports Med*. 2020;54:1055–1057. 32265218 10.1136/bjsports-2019-101343

