# Supplementary material for: What Personal and Work-Related Characteristics of Dutch Construction Workers With Knee Osteoarthritis Are Associated With Future Work Ability?
Source: J Occup Environ Med. 2022 Oct 13;65(3):271–6. doi: 10.1097/JOM.0000000000002730 (PMC9988233; doi:10.1097/JOM.0000000000002730)
Supplement: SUPPLEMENTARY MATERIAL [file joem-65-0271-s001.docx]

Abstract

**Objective**

To assess personal and work-related characteristics of construction workers with knee osteoarthritis (KO) associated with their ability to perform their current profession in the following two years.

**Methods**

A cross-sectional study was performed among Dutch construction workers diagnosed with KO using data from the Worker Health Surveillance (WHS). Logistic regression was used to assess the characteristics associated with future work ability.

**Results**

Based on 344 construction workers with KO, being able to perform their current profession in two years’ time was associated with working weekly 36-45 hours (OR=3.0-6.3), performing high-intensity exercises 1-2 times weekly (OR=2.0-2.6), age<56 years (OR=0.2-0.3) and not performing strenuous work activities like lifting and kneeling (OR=0.4-0.5).

**Conclusions**

To keep construction workers with KO at work, intervention studies should evaluate the effects of reducing strenuous work activities and promote leisure-time exercise.

**Key words:** prognosis**,** work ability, knee osteoarthritis; physical activity, prevention, occupational health surveillance

Introduction

The construction industry is characterized by high physical work demands and prevalent work-related musculoskeletal disorders (MSDs) (1-3). A review including a meta-analysis showed that knee complaints are the most common complaint in the construction industry second only to low back pain, with a one-year prevalence of 37% (95% CI 22–52) (4). These knee complaints are in large part attributable to knee osteoarthritis (KO), given the high prevalence of this work-related disease compared to other diagnoses like meniscal tears or bursitis (5-7). In the upcoming years, a steep rise in KO is forecasted, especially among workers aged between 40-65 years (8). Specific construction professions with a high risk of KO are floor layers, asphalt workers, sheet-metal workers, rock workers, plumbers, bricklayers, wood workers and concrete workers (9).

Given the high prevalence of KO and the high physical work demands, these construction workers deserve timely and effective occupational health care to maintain their work ability despite the debilitating effect of KO. Doctor-diagnosed KO has an almost twofold increased risk of sick leave and about 40-50% increased risk of disability pension compared with the general population (10). In addition, a recent Dutch study showed that the annual sick leave costs due to KO in the Dutch workforce are substantial for workers consulting an occupational physician, with estimated costs of $43 million. The average costs per sick leave episode are also considerable – $16,846 for KO with an average sick leave duration of 186 days (11). Unfortunately, no occupational health guidelines are available that address how work ability among workers with KO should be managed. Present clinical guidelines for non-operative treatment are primarily focused on pain and self-reported function of activities of daily life (12). These outcomes are not always a good proxy for being able to work (13).

To prevent sick leave and early retirement, and to contribute to a life span approach for KO, occupational health care should target prognostic factors that enhance the construction workers’ future work ability (14). Therefore, the aim of this study is to assess the personal and work-related characteristics of construction workers diagnosed with KO associated with their ability to perform their current profession in the following two years.

Methods

***Study design***

A cross-sectional study was conducted to determine the association between personal and work-related characteristics and the self-reported future ability of construction workers with KO to practice their current profession in two years’ time. The Strengthening the Reporting of Observational Studies in Epidemiology (STROBE) checklist was used to describe this cross-sectional study (Appendix 1, <http://links.lww.com/JOM/B219>)(15).

***Participants and measurement***

For this study, male construction workers aged between 20 and 68 years were selected. Participants were included when they received a KO diagnosis by an occupational physician. Data were used from the Worker Health Surveillance (WHS) for construction workers performed between 2011-2021 in the Netherlands. The WHS consists of two parts, consisting of: (1) a standardized physical examination; and (2) a self-administered questionnaire. Construction workers were invited to participate in the WHS starting at the age of 20 years, followed by periodic check-ups every five years and more regularly after the age of 50.

***Variables***

For the purpose of this study, specific questions from the WHS were selected focusing on knee complaints and future work ability (Appendix 2, <http://links.lww.com/JOM/B220>). Questions were selected based on risk factors from the literature for KO and work ability (16-35).

***Study size***

For this study, all construction workers were included if doctor-diagnosed KO was established. Data from the WHS between 2011 and 2021 were used, which resulted in a total sample size of 344 male construction workers with clinically-diagnosed KO (Figure 1).

*Figure 1 here.*

***Primary outcomes***

The selected primary outcome was the self-reported prognosis of work ability to practice the current profession in two years’ time measured with a self-administered questionnaire taken from the Work Ability Index questionnaire (36, 37). The answering categories were unlikely, maybe, and very likely. For this study, the population was divided into two groups: 1) construction workers who reported being ‘very likely to be practicing their current profession in two years’ time’ (answering category ‘very likely’); and 2) construction workers who reported being ‘not very likely to be practicing their current profession in two years’ time’ (answering categories ‘maybe’ and ‘unlikely’). The two groups were created to form comparable group sizes in the number of participants. Independent variables were age, Body Mass Index (BMI, body height and body weight were measured during the physical examination), number of years in current profession, average number of working hours per week, number of times per week of high-intensity exercise, performance of strenuous work activities and the worker being of the opinion that work caused the complaints. Continuous variables were recoded into categories. Age was recoded into four categories (< 40 – 49 years, 50 – 55 years, 56 – 60 years, > 60 years). Given the increased risk of KO with older age, in our opinion this categorization best represented the included participant group. BMI was categorized as normal weight (BMI 18-25 kg/m^2^), overweight (BMI 25-30 kg/m^2^), and obesity (BMI >30 kg/m^2^), number of years in current profession as 0 – 10 years, 11 – 20 years, 21 – 30 years, 31 – 40 years and 41 – 50 years, average number of working hours per week as 3 – 35 hours, 36 – 40 hours, 41 – 45 hours and >45 hours, number of times per week of high-intensity exercise as 0 times, 1 time, 2 times, and 3 times, performing regularly strenuous work activities defined as: 1) bending over; 2) kneeling or squatting; and/or 3) lifting, pushing, pulling, or carrying heavy loads during work with the answering categories of no strenuous work activity, one of these strenuous work activities, two of these activities, and three of these activities) and finally work ability on a scale from 0-10 categorized as 0 – 5 being poor, 6 – 7 being moderate, 8 – 9 being good, and 10 being excellent.

***Statistical methods***

To get insight into the association between personal and work-related characteristics and the future ability to practice the current profession in two years’ time, a binary logistic regression was conducted. Firstly, univariable analysis was conducted. Secondly, a multivariable binary logistic regression model was performed based on the significance of variables at *p* ≤ 0.05 from the univariable analysis. For the multivariable regression model, the forward stepwise procedure was used. Before performing the regression analysis, the included variables were tested on collinearity and power (Appendix 3, <http://links.lww.com/JOM/B221>). The quality of the model was assessed with the -2-log likelihood ratio test and the Hosmer-Lemeshow test (Appendix 4, <http://links.lww.com/JOM/B222>, and 5, <http://links.lww.com/JOM/B223>). A Hosmer-Lemeshow chi-squared with *p* ≥ 0.05 showed goodness of fit. For the results, categorical variables were presented as numbers and percentages. Numerical variables were additionally presented as a mean and their standard deviation (SD). The results of the logistic regression analysis were presented using Odds Ratio (OR) and their corresponding 95% confidence interval (95% CI), and the p-value was set at *p* ≤ 0.05. Data were analyzed using IBM SPSS Statistics version 26.

Results

***Participants and descriptive data***

As already stated, a total of 344 Dutch construction workers diagnosed with KO were included in this study (Figure 1). The personal and work-related characteristics of the participants are presented in Table 1. Most of the participants were above the age of 56 years, felt pain or stiffness in their knee, were overweight, and performed high-intensity exercise zero times per week. Moreover, they worked 36 – 40 hours per week and had worked in their current profession for 21 – 30 years. Participants reported having good physical and mental work ability and reported regularly bending over, kneeling or squatting, and lifting, pushing, pulling or carrying heavy loads during work.

*Table 1 here.*

***Personal and work-related characteristics of work ability in two years’ time***

The univariable binary logistic regression was performed first for the seven selected variables separately (Table 2). The two variables BMI and whether work had caused complaints were not significantly associated with work ability in two years’ time (p=0.42 and p=0.55, respectively) and were thus not included in the multivariable analysis.

*Table 2 here****.***

The final model for the analysis on the association between personal and work-related characteristics on work ability in two years’ time are presented in Table 3 and included four variables. Personal and work-related characteristics associated with the ability to practice the current profession in two years’ time were 1-time high-intensity exercise per week (OR 2.62; 95%CI 1.12-6.15), 2-times per week of high-intensity exercise (OR 2.00; 95%CI 1.06-3.80), working for 36-40 hours per week (OR 3.01; 95%CI 1.36-6.67), working 41-45 hours per week (OR 6.25; 95%CI 2.06-18.99), performing two strenuous work activities (OR 0.45; 95%CI 0.22-0.95), performing three strenuous work activities (OR 0.40; 95%CI 0.18-0.88), age between 56 – 60 years (OR 0.22; 95%CI 0.09-0.54) and age >60 years (OR 0.28; 95%CI 0.11-0.69). The variable numbers of years working in the current profession was not significant and was thus not included in the final model. The Hosmer-Lemeshow test showed a goodness of fit (X^2^ = 4.538, p = 0.806).

*Table 3 here.*

Discussion

Our study found that the ability to practice the current profession in two years’ time is associated with high-intensity exercises once or twice a week, working for 36-45 hours per week, performing less than two strenuous work activities and being younger than 56 years.

The following findings appear in line with previous studies on the topic of having KO and enhancing work participation. For instance, being physically active outside work seems especially important for workers in physically strenuous jobs. Despite the fact that the guidelines of the World Health Organization (WHO) on physical activity and sedentary behavior make no distinction between occupational physical activity and leisure-time physical activity, the present results support the assumption that the so-called Goldilock Principle also applies for workers with KO (38). This is in line with the results of a recent evaluation of this principle for construction and health care workers, reporting that workers who spent more time on physical activity during leisure reported less musculoskeletal pain (39). Of course, being physically active in leisure time is easier said than done given that construction workers do not have a fixed workplace and therefore often have to travel back and forth from work, leaving little time and energy to exercise before or after work. The positive work ability results for remaining physically active in leisure time are also in line with the non-operative treatment guidelines for KO, supporting a physically active lifestyle to enhance participation (40). Moreover, the Good Life with osteoArthritis in Denmark (GLA:D™) showed that regular intense exercise (twice weekly for 6 weeks) appeared to improve work participation, reducing sickness absence in one year from 24% (95%CI 21-28) to 15% (95%CI 12-18) (41).

Not surprisingly, a high physical workload due to frequent bending over, kneeling or squatting, and lifting, pushing, pulling, or carrying heavy loads is not beneficial for the work ability of construction workers with KO. A high physical workload is an established risk factor for the onset or worsening of KO (14, 35). Moreover, KO limits the ability to perform these activities and therefore the negative association found with the future work ability of construction workers with KO is in line with the literature (31). The same line of reasoning applies for older age. The prevalence of KO is of course strongly related to an increase of age (14, 17). In our study, we decided to create a composite outcome variable of physical workload activities as we expect high correlation between these activities (42). However, we also conducted additional analysis of the physical workload activities separately (appendix 6, <http://links.lww.com/JOM/B224>). The results of the additional analysis showed that the work activity of bending over of the trunk in the univariate analysis was not found to be significant. This might also be expected given that KO is less likely to limit trunk activity compared to the other two more strongly associated activies that are more demanding for the lower extremities namelijk ‘kneeling or squatting’ and ‘lifting, pushin, pulling, or carrying heavy loads during work’.

Not all findings appear in line with previous studies, however. The fact that working less than 36 hours per week does not support future work ability appears contradictory to current findings (43). Working 36-45 hours appears optimal. However, continuing to work a high number of hours as a construction worker does not seem a wise decision, despite construction workers not being able to resist it. On the other hand, the result in this study can possibly be explained due to a *healthy worker’s survival effect,* which means that, in this study, construction workers with KO who were absent from work or who retired early did not participate in the surveillance (44). However, we believe that the risk of selection bias is minimal in our study (45). Both the two oldest age groups (56 – 60 years and >60 years) are less likely to do their current profession in two years’ time compared to their younger colleageus (see table 3). This is problably explained by the fact that KO is a progressive disease for which no cure available is (16, 17).

It is interesting to note that BMI was not found to be associated with a less favorable prognosis of work ability in two years’ time (46). This is counterintuitive given that BMI is associated with an increased risk of KO (14)and having a higher BMI as a KO patient results in a higher pain score (47). A possible explanation is that workers with KO and being overweight or obese have already have structured their job in such a manner that they no longer need to perform the most demanding activities, given their body weight. Further research should be done to properly determine the influence of BMI on the prospects of the further career of the construction workers with KO. Moreover, the use of BMI has been debated in the literature as controversial (48). Unless the fact that BMI might not be the most accurate and suitable measure for determining overweight or obesity, it is one of the most used measurements to screen overweight or obesity risks in various population groups and it is well suitable for epidemiological settings such as the correlation between health outcomes and BMI (49). Therefore, measurement of BMI was deemed appropriate for the purpose of the current study, but future studies should investigate the influence of body composition on future work ability especially in physically demanding jobs.’’

In order to offer tailor-made prevention and interventions for male construction workers with KO, future research should focus on reducing physically demanding work activities and promoting high-intensity exercise for at least once a week. Previous research among construction workers showed that health promotion worksite interventions improved physical activity and may, thus, contribute to a higher ability to practice their current profession in the future (50). In addition, a Total Worker Health® Intervention among construction workers showed that at six months, the intervention group had reduced physically demanding activities and increased recreational physical activity (51). Unfortunately, limited evidence is available to support the suggestion that the use of preventive ergonomic measures to reduce the workload actually reduces the risk of associated musculoskeletal complaints (52). However, health impact assessments based on work site measurements potentially show positive gains of ergonomics measures at the work site among construction workers at risk of KO (53).

***Limitations and strengths***

A major strength of our study is the presence of a high number of construction workers diagnosed with KO by an occupational physician. Moreover, investigating the combination of personal and work-related characteristics can also be seen as a strength, since both factors influence the ability to continue working as a construction worker with KO. Finally, work ability is measured using a validated method (25).

Nevertheless, we faced three limitations in our study. Firstly, this study had a cross-sectional design that does not allow investigating the causal relationship between the outcome and variables (54). Secondly, the method of diagnosing KO used by the various occupational physicians might have differed. This could have resulted in a more heterogeneous selection of workers who did not have KO as the primary cause of their knee complaints. Moreover, participation in the WHS is voluntary, which might have resulted in a healthy worker selection effect (44). Reasons for not participating or having a lower commitment could be that construction workers were afraid that a negative outcome regarding their future career opportunities might result, or they did not see the personal relevance to participate (44). A third limitation was that the WHS questionnaire made no explicit distinction between occupational physical activity and leisure-time physical activity (55). This may have introduced classification bias as it is challenging to distinguish whether exercise concerns occupational physical activity or leisure time physical activity (55). An earlier study recommends physical activity assessment based on objective measures including, for example, accelerometers instead of self-reported questionnaire-based assessment (55). However, based on the results of the current study, we assume that participants considered the question on exercise as leisure-time physical activity as most participants reported zero times per week of high-intensity exercise.

Lastly, for the primary outcome measure the self-reported prognosis of work ability to practice the current profession in two years’ time, the category ‘maybe’ and ‘unlikely’ were combined to form a dichotomous outcome measure for the analysis. Combination of those categories might be conservative, but we aimed to study the prognostic factors that enhance the construction workers’ future work ability to prevent sick leave and early retirement targeting the group that may be at risk of being unable to do current profession in two years’ time and that believes to be certain to be unable to do current profession in two years’ time. Thus, the current study gives insight into the needs for tailoring future interventions to the prognostic factors of both the at-risk group as well as the group unlikely to do current profession in the future.

Conclusion

Dutch construction workers with clinically-diagnosed KO who reported that, in two years’ time, they would be able to practice their current job were working 36-45 hours per week, performing high-intensity exercises once or twice a week, were aged < 56 years, and performed less than two strenuous physical activities in their work compared to their counterparts who reported not being able to practice their current job in two years’ time. To keep construction workers with KO at work, intervention studies should evaluate the effects of reducing strenuous work activities and promote leisure-time exercise.

References

1. Colin R, Wild P, Paris C, Boini S. Effect of Joint Exposure to Psychosocial and Physical Work Factors on the Incidence of Workplace Injuries: Results From a Longitudinal Survey. Journal of Occupational and Environmental Medicine. 2021;63(11):921-30.

2. What are the Risks to Minors Who Work in the Construction Industry? Journal of Occupational and Environmental Medicine. 2021;63(7):e462-e3.

3. Dale AM, Rohlman DS, Hayibor L, Evanoff BA. Work Organization Factors Associated with Health and Work Outcomes among Apprentice Construction Workers: Comparison between the Residential and Commercial Sectors. International Journal of Environmental Research and Public Health. 2021;18(17):8899.

4. Umer W, Antwi-Afari MF, Li H, Szeto GPY, Wong AYL. The prevalence of musculoskeletal symptoms in the construction industry: a systematic review and meta-analysis. International Archives of Occupational and Environmental Health. 2018;91(2):125-44.

5. Hulshof CTJ, Pega F, Neupane S, Colosio C, Daams JG, Kc P, et al. The effect of occupational exposure to ergonomic risk factors on osteoarthritis of hip or knee and selected other musculoskeletal diseases: A systematic review and meta-analysis from the WHO/ILO Joint Estimates of the Work-related Burden of Disease and Injury. Environment International. 2021;150:106349.

6. Bahns C, Bolm-Audorff U, Seidler A, Romero Starke K, Ochsmann E. Occupational risk factors for meniscal lesions: a systematic review and meta-analysis. BMC Musculoskeletal Disorders. 2021;22(1):1042.

7. Le Manac'h AP, Ha C, Descatha A, Imbernon E, Roquelaure Y. Prevalence of knee bursitis in the workforce. Journal of Occupational and Environmental Medicine. 2012;62(8):658-60.

8. Kuijer P, Burdorf A. Prevention at work needed to curb the worldwide strong increase in knee replacement surgery for working-age osteoarthritis patients. Scandinavian Journal of Work, Environment and Health. 2020;46(5):457-60.

9. Jarvholm B, From C, Lewold S, Malchau H, Vingard E. Incidence of surgically treated osteoarthritis in the hip and knee in male construction workers. Journal of Occupational and Environmental Medicine. 2008;65(4):275-8.

10. Hubertsson J, Petersson IF, Thorstensson CA, Englund M. Risk of sick leave and disability pension in working-age women and men with knee osteoarthritis. Annals of the Rheumatic Diseases. 2013;72(3):401-5.

11. Hardenberg M, Speklé EM, Coenen P, Brus IM, Kuijer PPFM. The economic burden of knee and hip osteoarthritis: absenteeism and costs in the Dutch workforce. BMC Musculoskeletal Disorders. 2022;23(1):364.

12. Bannuru RR, Osani MC, Vaysbrot EE, Arden NK, Bennell K, Bierma-Zeinstra SMA, et al. OARSI guidelines for the non-surgical management of knee, hip, and polyarticular osteoarthritis. Osteoarthritis Cartilage. 2019;27(11):1578-89.

13. Van Zaanen Y, Hoorntje A, Koenraadt KLM, Van Bodegom-Vos L, Kerkhoffs G, Waterval-Witjes S, et al. Non-surgical treatment before hip and knee arthroplasty remains underutilized with low satisfaction regarding performance of work, sports, and leisure activities. Acta Orthopaedica. 2020;91(6):717-23.

14. Whittaker JL, Runhaar J, Bierma-Zeinstra S, Roos EM. A lifespan approach to osteoarthritis prevention. Osteoarthritis Cartilage. 2021;29(12):1638-53.

15. STROBE Checklists STROBE: Strengthening the reporting of observational studies in epidemiology [Available from: <https://www.strobe-statement.org/checklists/>.

16. Katz JN, Arant KR, Loeser RF. Diagnosis and Treatment of Hip and Knee Osteoarthritis: A Review. JAMA. 2021;325(6):568-78.

17. Shane Anderson A, Loeser RF. Why is osteoarthritis an age-related disease? Best Pract Res Clin Rheumatol. 2010;24(1):15-26.

18. Altman R, Asch E, Bloch D, Bole G, Borenstein D, Brandt K, et al. Development of criteria for the classification and reporting of osteoarthritis. Classification of osteoarthritis of the knee. Diagnostic and Therapeutic Criteria Committee of the American Rheumatism Association. Arthritis and Rheumatology. 1986;29(8):1039-49.

19. Stubbs B, Aluko Y, Myint PK, Smith TO. Prevalence of depressive symptoms and anxiety in osteoarthritis: a systematic review and meta-analysis. Age Ageing. 2016;45(2):228-35.

20. Toivanen AT, Heliövaara M, Impivaara O, Arokoski JPA, Knekt P, Lauren H, et al. Obesity, physically demanding work and traumatic knee injury are major risk factors for knee osteoarthritis—a population-based study with a follow-up of 22 years. Rheumatology. 2009;49(2):308-14.

21. Hubertsson J, Petersson IF, Thorstensson CA, Englund M. Risk of sick leave and disability pension in working-age women and men with knee osteoarthritis. Annals of the Rheumatic Diseases. 2013;72(3):401-5.

22. Wilkie R, Blagojevic-Bucknall M, Jordan KP, Lacey R, McBeth J. Reasons why multimorbidity increases the risk of participation restriction in older adults with lower extremity osteoarthritis: a prospective cohort study in primary care. Arthritis Care Res (Hoboken). 2013;65(6):910-9.

23. Connelly AE, Tucker AJ, Kott LS, Wright AJ, Duncan AM. Modifiable lifestyle factors are associated with lower pain levels in adults with knee osteoarthritis. Pain Research and Management. 2015;20(5):241-8.

24. Zheng H, Chen C. Body mass index and risk of knee osteoarthritis: systematic review and meta-analysis of prospective studies. BMJ Open. 2015;5(12):e007568.

25. Łastowiecka E, Bugajska J, Najmiec A, Rell-Bakalarska M, Bownik I, Jędryka-Góral A. Occupational work and quality of life in osteoarthritis patients. Rheumatology International. 2006;27(2):131-9.

26. Kujala UM, Kettunen J, Paananen H, Aalto T, Battie MC, Impivaara O, et al. Knee osteoarthritis in former runners, soccer players, weight lifters, and shooters. Arthritis and Rheumatology. 1995;38(4):539-46.

27. Jarvholm B, Lewold S, Malchau H, Vingard E. Age, bodyweight, smoking habits and the risk of severe osteoarthritis in the hip and knee in men. European Journal of Epidemiology. 2005;20(6):537-42.

28. Järvholm B, From C, Lewold S, Malchau H, Vingård E. Incidence of surgically treated osteoarthritis in the hip and knee in male construction workers. Journal of Occupational and Environmental Medicine. 2008;65(4):275-8.

29. Holmberg S, Thelin A, Thelin N. Is there an increased risk of knee osteoarthritis among farmers? A population-based case–control study. International Archives of Occupational and Environmental Health. 2004;77(5):345-50.

30. Andersen S, Thygesen LC, Davidsen M, Helweg-Larsen K. Cumulative years in occupation and the risk of hip or knee osteoarthritis in men and women: a register-based follow-up study. Journal of Occupational and Environmental Medicine. 2012;69(5):325-30.

31. McWilliams DF, Leeb BF, Muthuri SG, Doherty M, Zhang W. Occupational risk factors for osteoarthritis of the knee: a meta-analysis. Osteoarthritis Cartilage. 2011;19(7):829-39.

32. Manninen P, Heliovaara M, Riihimaki H, Suoma-Iainen O. Physical workload and the risk of severe knee osteoarthritis. Scandinavian Journal of Work, Environment and Health. 2002;28(1):25-32.

33. Jones GT, Harkness EF, Nahit ES, McBeth J, Silman AJ, Macfarlane GJ. Predicting the onset of knee pain: results from a 2-year prospective study of new workers. Annals of the Rheumatic Diseases. 2007;66(3):400-6.

34. Kievit AJ, van Geenen RC, Kuijer PP, Pahlplatz TM, Blankevoort L, Schafroth MU. Total knee arthroplasty and the unforeseen impact on return to work: a cross-sectional multicenter survey. The Journal of Arthroplasty. 2014;29(6):1163-8.

35. Verbeek J, Mischke C, Robinson R, Ijaz S, Kuijer P, Kievit A, et al. Occupational Exposure to Knee Loading and the Risk of Osteoarthritis of the Knee: A Systematic Review and a Dose-Response Meta-Analysis. Safety and Health at Work. 2017;8(2):130-42.

36. Ilmarinen J. The Work Ability Index (WAI). Journal of Occupational and Environmental Medicine. 2007;57(2):160-.

37. de Zwart BC, Frings-Dresen MH, van Duivenbooden JC. Test-retest reliability of the Work Ability Index questionnaire. Journal of Occupational and Environmental Medicine. 2002;52(4):177-81.

38. WHO guidelines on physical activity and sedentary behaviour. World Health Organization; 2020.

39. Merkus SL, Coenen P, Forsman M, Knardahl S, Veiersted KB, Mathiassen SE. An Exploratory Study on the Physical Activity Health Paradox&mdash;Musculoskeletal Pain and Cardiovascular Load during Work and Leisure in Construction and Healthcare Workers. International Journal of Environmental Research and Public Health. 2022;19(5):2751.

40. Jayabalan P, Ihm J. Rehabilitation Strategies for the Athletic Individual with Early Knee Osteoarthritis. Current Sports Medicine Reports. 2016;15(3):177-83.

41. Skou ST, Roos EM. Good Life with osteoArthritis in Denmark (GLA:D™): evidence-based education and supervised neuromuscular exercise delivered by certified physiotherapists nationwide. BMC Musculoskeletal Disorders. 2017;18(1):72.

42. Manninen P, Heliovaara M, Riihimaki H, Suoma-Iainen O. Physical workload and the risk of severe knee osteoarthritis. Scand J Work Environ Health. 2002;28(1):25-32.

43. Iliades C. Managing Osteoarthritis at Work. Everyday Health 2009.

44. Siebert U, Rothenbacher D, Daniel U, Brenner H. Demonstration of the healthy worker survivor effect in a cohort of workers in the construction industry. Journal of Occupational and Environmental Medicine. 2001;58(12):774-9.

45. Chowdhury R, Shah D, Payal AR. Healthy Worker Effect Phenomenon: Revisited with Emphasis on Statistical Methods - A Review. Indian J Occup Environ Med. 2017;21(1):2-8.

46. Robroek SJW, Järvholm B, van der Beek AJ, Proper KI, Wahlström J, Burdorf A. Influence of obesity and physical workload on disability benefits among construction workers followed up for 37 years. Journal of Occupational and Environmental Medicine. 2017;74(9):621-7.

47. Raud B, Gay C, Guiguet-Auclair C, Bonnin A, Gerbaud L, Pereira B, et al. Level of obesity is directly associated with the clinical and functional consequences of knee osteoarthritis. Scientific Reports. 2020;10(1):3601.

48. Frankenfield DC, Rowe WA, Cooney RN, Smith JS, Becker D. Limits of body mass index to detect obesity and predict body composition. Nutrition. 2001;17(1):26-30.

49. Claessen H, Arndt V, Drath C, Brenner H. Overweight, obesity and risk of work disability: a cohort study of construction workers in Germany. Occup Environ Med. 2009;66(6):402-9.

50. Viester L, Verhagen EALM, Bongers PM, van der Beek AJ. The effect of a health promotion intervention for construction workers on work-related outcomes: results from a randomized controlled trial. International Archives of Occupational and Environmental Health. 2015;88(6):789-98.

51. Peters SE, Grant MP, Rodgers J, Manjourides J, Okechukwu CA, Dennerlein JT. A Cluster Randomized Controlled Trial of a Total Worker Health® Intervention on Commercial Construction Sites. International Journal of Environmental Research and Public Health. 2018;15(11):2354.

52. van der Molen HF, Sluiter JK, Frings-Dresen MH. The use of ergonomic measures and musculoskeletal complaints among carpenters and pavers in a 4.5-year follow-up study. Ergonomics. 2009;52(8):954-63.

53. Visser S, Van der Molen H, Kuijer P. A health impact assessment of a preventive measure to reduce the risk of work-related low back pain, lumbosacral radiculopathy and knee osteoarthritis among construction workers in the Netherlands. Safety and Health at Work. 2022;13:S145.

54. Kestenbaum B. Cross-Sectional Studies. Epidemiology and Biostatistics: Practice Problem Workbook. Cham: Springer International Publishing; 2019. p. 9-11.

55. Coenen P, Huysmans MA, Holtermann A, Krause N, van Mechelen W, Straker LM, et al. Towards a better understanding of the ‘physical activity paradox’: the need for a research agenda. British Journal of Sports Medicine. 2020;54(17):1055-7.

Figure legend

***Figure 1:*** *Flowchart - inclusion and exclusion criteria study participants.*

Table legend

***Table 1:*** *Descriptive personal and work-related characteristics of construction workers with KO.*

***Table 2****: Univariate analysis of the association* *(odds ratio, 95% confidence interval, p-value) between personal and work-related characteristics and ability to practice the current profession in two years’ time.*

***Table 3:*** *Multivariable analysis* *of the association (odds ratio, 95% confidence interval, p-value) between personal and work-related characteristics and ability to practice the current profession in two years’ time.*

List of supplemental digital content:

Appendix 1.docx

Appendix 2.docx

Appendix 3.docx

Appendix 4.docx

Appendix 5.docx

Appendix 6.docx
